# NNRTI-induced HIV-1 protease-mediated cytotoxicity induces rapid death of CD4 T cells during productive infection and latency reversal

**DOI:** 10.1186/s12977-019-0479-9

**Published:** 2019-06-26

**Authors:** Benjamin Trinité, Hongtao Zhang, David N. Levy

**Affiliations:** 1 Department of Basic Science, New York University College of Dentistry, New York, NY USA; 2 Present Address: IrsiCaixa AIDS Research Institute, Badalona, Spain

**Keywords:** HIV, NNRTI, Protease inhibitor, Cure therapy, Latent reservoir

## Abstract

**Background:**

Current efforts towards HIV-1 eradication focus on the reactivation and elimination of the latent viral reservoir, so-called shock and kill therapy. However, work from several groups indicates that infected cell death following virus reactivation is not guaranteed. Thus, it is imperative to develop strategies to foster specific elimination of cells carrying integrated proviruses. It has been shown that some non-nucleoside reverse transcriptase inhibitors (NNRTIs) including efavirenz can induce premature HIV-1 GagPol dimerization in productively infected cells, resulting in intracellular HIV-1 Protease (PR) activation and a reduction in HIV-1 expressing cells.

**Results:**

Here, we document that NNRTI-induced PR activation triggers apoptotic death of productively infected resting or activated T cells in as little as 2 h via caspase-dependent and independent pathways. Rilpivirine, efavirenz and etravirine were the most potent NNRTIs, whereas nevirapine had almost no effect. NNRTI-induced cell killing was prevented by inhibitors of HIV-1 Protease (PR) activity including indinavir and nelfinavir. HIV-1 transmitter founder viruses induced cell killing similarly to lab-adapted HIV-1 except when NNRTI resistance conferring mutations were present in reverse transcriptase. Mutations in PR that confer PR inhibitor (PI) resistance restore NNRTI-induced killing in the presence of PI. Finally, we show that NNRTIs can rapidly eliminate cells in which latent viruses are stimulated to active expression.

**Conclusions:**

This work supports the notion that select NNRTIs might help promote the elimination of HIV-1 producing cells as an adjuvant during shock and kill therapy.

**Electronic supplementary material:**

The online version of this article (10.1186/s12977-019-0479-9) contains supplementary material, which is available to authorized users.

## Background

Current efforts for HIV-1 eradication focus on the reactivation and elimination of the latent viral reservoir. The so-called “shock and kill” strategy envisages pharmacologically-induced activation of latently infected cells resulting in infected cell death by viral cytotoxicity or cellular immunity. However, evidence indicates that the cells expressing reactivated viruses do not necessarily die [1–4]. Therefore, additional co-treatments that specifically induce the death of virus-expressing cells may be necessary for the elimination of viral reservoirs.

HIV-1 Reverse Transcriptase (RT) and Protease (PR) are initially translated as part of the GagPol polyprotein. Contemporaneous with viral budding, PR autocatalytically cleaves GagPol, liberating itself and the other Gag and Pol subunits to generate the mature infectious viral particle. Activation of PR after rather than before virion assembly is likely a strategy to avoid improper targeting of PR’s catalytic activity to cytosolic host cell proteins. Antiviral Protease inhibitors (PIs) such as indinavir prevent generation of infectious viruses by blocking PR-dependent virus maturation.

Like PIs, non-nucleoside reverse-transcriptase inhibitors (NNRTIs) are also an important class of anti-HIV-1 drugs, but NNRTIs, as well as nucleoside RT inhibitors, block HIV-1 replication at an early stage of infection. NNRTIs bind to RT that has been delivered into cells within the infecting virion, where they sterically inhibit reverse transcription, the conversion of the viral RNA genome into a DNA copy that integrates into the host cell genome. In this way NNRTIs prevent de novo infection of cells by acting at an early stage in the viral replication cycle, whereas PIs only prevent spread of virus from already-infected cells by acting at the late stage of virus maturation.

However, when NNRTIs are present within cells that are already infected and are producing viral proteins, they may bind to the RT portion of a newly translated GagPol polyprotein and promote its homodimerization within the cytoplasm of the cell [5–7]. This results in premature PR activation independent of virus assembly and lead to a decrease in viral production [8]. Similar premature PR activation and decrease in virus production has been observed following overexpression of GagPol [9, 10]. PR activity within the host cytoplasm can lead to non-specific cleavage of multiple host proteins, including proteins that induce cytotoxic effects such as apoptosis [11–14]. Therefore, NNRTI-induced premature HIV-1 PR activation during a late stage of virus replication could provide a new strategy to help eliminate infected T cells. This idea was first explored by The Muller laboratory, which showed that after 3–5 days of treatment with select NNRTIs, the proportion of HIV-1 expressing MT4 cells (a transformed T cell line) and activated PBMCs was reduced [15]. The Sluis-Cremer laboratory also showed that during latency reversal, addition of NNRTIs was associated with a reduction in infected cells and virus output [16].

Here, we directly demonstrate that NNRTIs can induce apoptotic death of HIV-1 -infected resting or activated primary CD4 T cells. Bystander cells not expressing HIV-1 proteins were not affected. HIV-1 PR-dependence of this killing was demonstrated by complete prevention of NNRTI-dependent killing by PIs except when PI resistance conferring mutations were present in PR. Importantly, NNRTI-induced cell death was very rapid, suggesting that short term in vivo high dose NNRTI treatment might effectively eliminate productively infected cells. Finally, NNRTIs were effective in killing latently infected cells following latency reversal, suggesting that NNRTI treatment could be beneficial as an adjuvant to the kill portion of shock and kill therapy.

## Results

### NNRTI induce HIV-1 PR-dependent death of productively infected resting and activated CD4 T cells

**Fig. 1:**
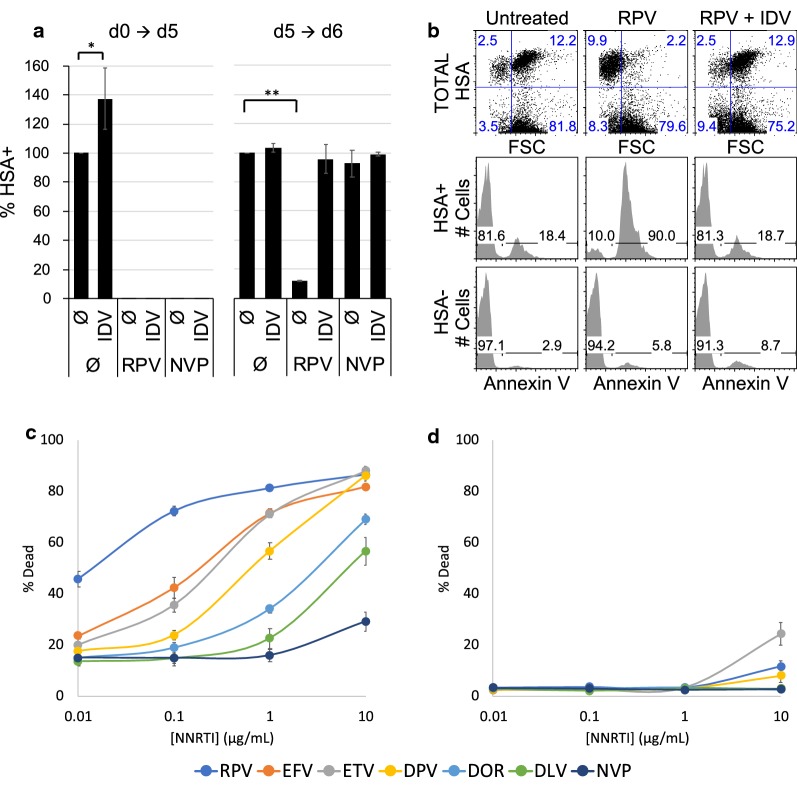
NNRTI treatment induces the death of productively HIV-1 infected cells. **a**–**d** Resting CD4 T cells were infected with a single round HSA reporter HIV-1 virus and incubated with IL-7 (2 ng/mL). **a** Cells were treated from 0 dpi (day post-infection) to 5 dpi or from 5 to 6 dpi with 1 μM of RPV, IDV and/or NVP as indicated. At 5 dpi and 6 dpi respectively, cells were stained for HSA and analyzed by flow cytometry. Histograms show the percentage of HSA+ cells detected among morphologically live cells (determined using FSC and SSC) and normalized to the untreated group in each graph. Data are averages and SD of 3 cell donors and are representative of 3 or more independent experiments. (*p = 0.0409; **p < 0.0001: p-values were calculated with an unpaired two-tailed t-test). **b** At 5 dpi infected cells were treated with 1 μM of RPV. Twenty-four hours later, cells were stained for HSA, labeled with Annexin V and analyzed by flow cytometry. Upper panels are dot plots showing HSA expression vs. cell size parameter (based of FSC). Lower panels show Annexin V labeling in pre-gated HSA+ and HSA negative cells. Data are representative of 3 or more independent experiments. **c**, **d** Infected cells were treated at 5 dpi with various concentrations of the indicated NNRTIs. Twenty-four hours later cells were analyzed for survival using FSC and SSC. Data are averages and SD of 3 donors and are representative of 2 independent experiments. **c** Survival of HSA+ cells. **d** Survival of HSA-negative cells

Owing to the observation that NNRTI-induced HIV-1 PR activation could induce a reduction in the number of productively infected activated PBMCs [15], we tested whether NNRTI could induce specific killing of resting CD4 T cells. We employed a reporter virus that expresses the surface antigen Heat Stable Antigen (HSA, mCD24) [17], as this protein remains detectable on dying cells longer than intracellular fluorescent proteins (Additional file 1: Fig. S2). Infection was held to a single round by mutation of the *env* gene, preventing the generation of infectious virions in target cells. Single round infection of resting T cells [18] achieves maximal expression around day 5 [19]. We treated cells with 1 μM of the NNRTI rilpivirine (RPV) or nevirapine (NVP) either on the day of infection (d0) to block reverse transcription [20], or on day 5 to test cell killing. Both RPV and NVP were effective at blocking productive infection of resting T cells when added, on d0, prior to reverse transcription initiation (Fig. 1a). Interestingly, when added on d5, RPV but not NVP resulted in a steep reduction of HIV-1 expressing cells by d6. Loss of HSA+ cells was completely prevented by the PI indinavir (IDV), suggesting that HIV-1 PR activity was required for cell killing, consistent with the data from Jochmans et al. [15]. While no HSA+ cells were detected when reverse transcription was inhibited (d0 treatment), dead HSA+ cells were detected on d5, evidenced by a reduced forward scatter profile (Fig. 1b). Cell death was confirmed by labeling with Annexin V staining of HSA+ cells but was not increased on HSA-negative cells that were not productively infected. Reduction in forward scatter and increased Annexin V staining were both abolished by IDV treatment. Interestingly when IDV was added on the day of infection, productive infection measured at 5-days post infection (dpi) was increased, suggesting a protective role of IDV against spontaneous viral cytotoxicity.

We then tested several NNRTIs for their ability to induce death of HIV-1 expressing cells. RPV, efavirenz (EFV) and etravirine (ETV) stood out as the most effective (Fig. 1c). Slight toxicity on HSA− cells (Fig. 1d) and mock infected cells (Additional file 1: Fig. S3) was observed at 10 μM but remained far below the levels of killing of HIV-expressing cells.

**Fig. 2:**
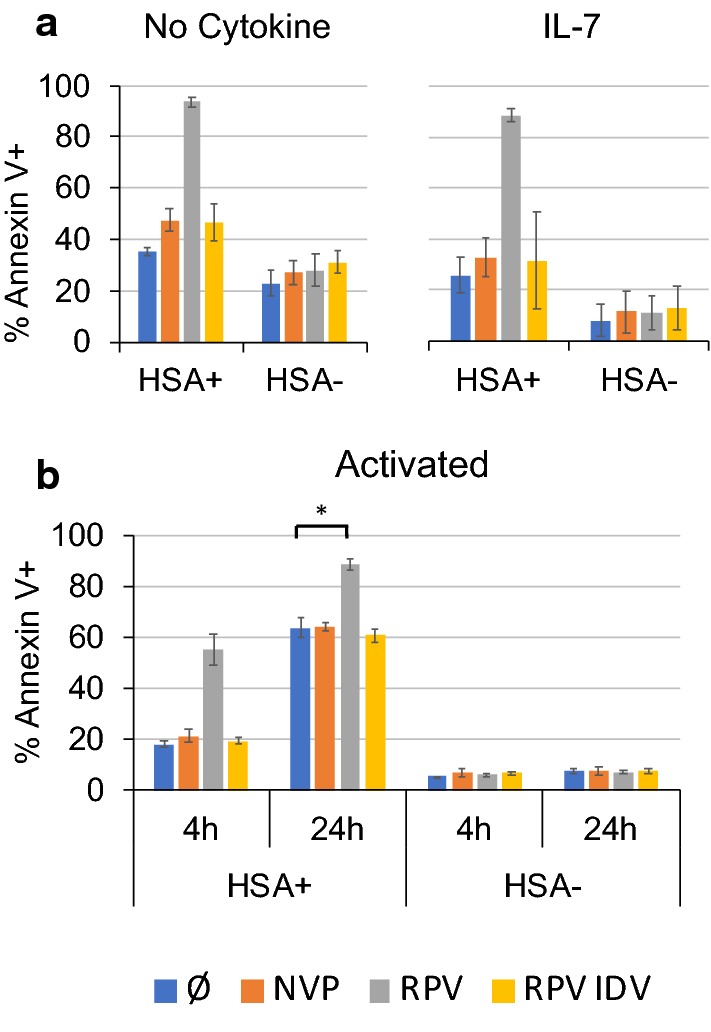
Both resting and activated T cells are sensitive to NNRTI-induced killing. **a**, **b** Comparison of resting T cells treated or not with IL-7 (**a**) and anti-CD3/CD28 activated T cells (infected 2 days after activation) (**b**). At 5 dpi (resting) or 2 dpi (activated) cells were treated or not with RPV, NVP and or IDV (1 μM each) and stained 4 h (activated) and 24 h (resting and activated) later, with annexin V and anti-HSA antibody. Data indicate Annexin V labeling in pre-gated HSA+ and HSA− fractions. Data are averages and SD of 3 donors and representative of 2 independent experiments. (*p = 0.0006: p-value was calculated with an unpaired two-tailed t-test)

We then compared the efficacy of this treatment on 3 different cell models: IL-7 treated resting T cells as above, non-cytokine treated resting T cells, and activated T cells. We have previously shown that common gamma chain cytokines such as IL-7 block the death of resting T cells resulting from reverse transcription and Vpr-induced toxic effects [20]. Resting T cells were treated on d5 after infection and activated T cells were treated on d2 after infection. In line with our previous work, spontaneous cell death was increased in absence of cytokine in both HSA+ and HSA− cells. Nevertheless, RPV- (Fig. 2) and EFV-induced (Additional file 1: Fig. S4) killing were effective in both cytokine-treated and non-treated T cells, indicating that IL-7 did not inhibit cell death by this mechanism. Furthermore, the addition of additional IL-7 at the time of RPV treatment provided no protection (Additional file 1: Fig. S5). In the case of activated T cells, spontaneous viral toxicity was significant in virus expressing (HSA+) cells, leading to the killing of about 60% of the productively infected cells within 24 h, even in the absence of NNRTI. RPV treatment did, however, further increased this cell death to about 90%. Interestingly, in both resting and activated T cells, the presence of IDV protected activated T cells from death induced by the NNRTI treatment but not by normal viral cytopathy, suggesting that in this case virus toxicity does not depend on PR activity. In all cases, HSA-negative cells were not affected by NNRTI treatment.

### NNRTI induce rapid caspase-dependent and caspase-independent cell death of HIV-1 expressing T cells

**Fig. 3:**
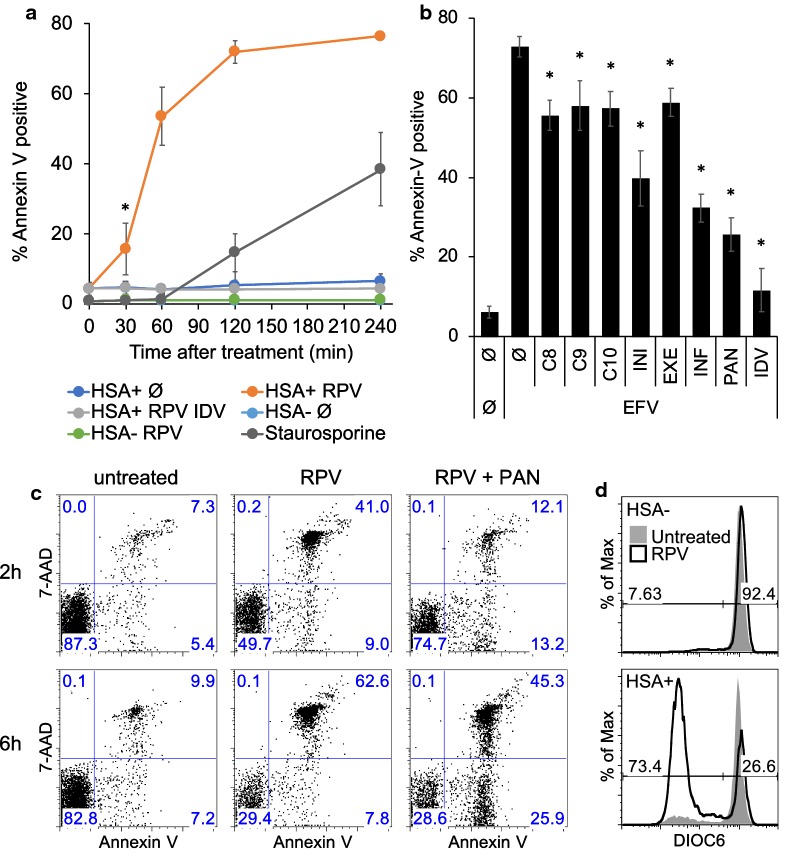
NNRTI-induced killing is rapid and occurs via caspase-dependent and caspase-independent pathways. **a** Resting CD4 T cells were infected with single round HSA reporter HIV-1 virus and incubated with IL-7. At 5 dpi, cells were treated with 1 μM of IDV and/or RPV. Cells were harvested at the indicated times and kept on ice until all the time points were harvested. Cells were then stained for HSA, labeled with Annexin V and analyzed by flow cytometry. Uninfected control cells were treated with staurosporine. (*p = 0.0281: p-value was calculated at 30′, between RPV HSA+ and RPV HSA− populations, with an unpaired two-tailed t-test). Data are averages and SD of 3 cell donors and are representative of 3 independent experiments. **b** At 5 dpi, HSA virus infected cells were treated or not with a panel of caspase inhibitors (as indicated, see “Methods”) for 1 h and then treated with EFV (1 μM) for 2 h. Cells were then analyzed for Annexin V labeling and HSA expression. Histogram indicates the percentage of Annexin V positive cells within the HSA+ fraction. Data are averages and SD of 3 donors and are representative of 3 independent experiments. INI = initiator caspases 8, 9, 10; EXE = executioner caspases 3 and 6; INF = inflammatory caspases 1 and 13. PAN = pan-caspase inhibitor Z-VAD-FMK. All the inhibition were statistically different from EFV alone (p < 0.05), indicated by “*”. Inhibition with INI cocktail was statistically different from the effect of the individual caspase inhibitors 8, 9 and 10 (p < 0.05). (p-values were calculated with an unpaired two-tailed t-test). **c** HSA reporter virus infected cells were treated at 5 dpi with RPV (1 μM) for 2 and 6 h in the presence of Z-VAD-FMK (PAN) or not and stained for HSA expression and with Annexin V and 7-AAD. Dot plots show Annexin V and 7-AAD labeling within pre-gated HSA+ cells. Data are from 1 donor and are representative of 2 independent experiments. **d** HSA reporter virus infected cells were treated at 5 dpi with RPV (1 μM) for 2 h and stained for HSA expression and with DioC6. Data are 1 donor and representative of 2 independent experiments

We then examined the kinetics of NNRTI-dependent killing. Surprisingly, Annexin V labeling was detected within 30 min of NNRTI treatment (Fig. 3a) and was found on about half of the HIV-1 expressing T cells within 1 h. This fast induction contrasts sharply with the kinetics of staurosporine, commonly used in research as an inducer of apoptosis.

Previous reports have demonstrated that cytosolic PR activity targets several host proteins including caspase 8 [21, 22] as well as members of the mitochondrial apoptotic pathway [12, 23]. We therefore tested a panel of caspase inhibitors for their ability to block NNRTI-induced killing. Individual inhibitors for initiator caspases 8, 9 and 10 were only partially able to reduce cell death, while combinations were more effective (Fig. 3b). Combined inhibitors of executioner caspase 3 and 6 were also partially effective, as were combinations of inhibitors of the pro-inflammatory caspase 1, 4 and 13. Pan-caspase inhibitor Z-VAD-FMK (PAN) was the most potent inhibitor (Fig. 3b). Importantly, Z-VAD-FMK merely delayed cell death but did not ultimately prevent it. By 6 h, Z-VAD-FMK treated cells displayed similar Annexin-V labeling as cells treated only with RPV (Fig. 3c), although the fraction of Annexin-V +/7-AAD− cells was higher, indicating slower kinetics. Despite the rapid cell death, the sequential appearance of Annexin-V staining followed by 7-AAD staining indicates apoptosis (Fig. 3c). Finally, we also observed early mitochondrial depolarization (Fig. 3d) and Cytochrome c release (not shown). These results suggest, in accordance with previous studies, that PR is probably directly targeting multiple components of the apoptosis machinery. The direct activation of the suicide machinery rather than the induction of initial cellular stress signals may explain the rapidity of the cell death observed.

### Mutations conferring resistance to NNRTI inhibition of reverse transcription also confer resistance to NNRTI-induced killing

**Fig. 4:**
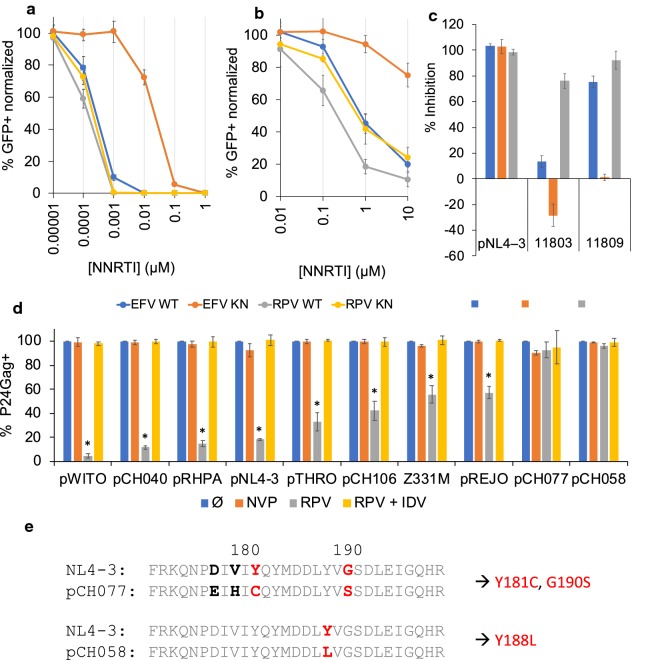
NNRTI-induced killing is sensitive to HIV-1 mutations leading to both NNRTI and PI resistance. **a**, **b** Cells were infected with single round GFP reporter virus containing or not the NNRTI resistant conferring RT K103N mutation. Cells were treated with the indicated dose of EFV and RPV on day 0 (**a**) or day 5 (**b**) and analyzed 5 days and 4 h later, respectively. The % of GFP+ cells within the live cells (defined by FSC and SSC), was normalized to the untreated condition for the corresponding virus (= 100%). **c** Inhibition NNRTI-induced killing of WT and PI resistant HIV-1 by PIs. Cells were infected with NL4-3 virus or patient isolated viruses containing a PI resistant PR. At 5 dpi cells were treated with RPV (1 μM) and the PR inhibitors IDV (1 μM), SQV (1 μM) or TPV (2 μM) and samples were collected 4 h later. Productively infected cells were identified by intracellular p24Gag staining within the live cells (defined by FSC and SSC). **d** NNRTI killing tested on a panel of transmitter/founder (TF) viruses. All the viruses were sensitive to NNRTI killing except pCH058 and pCH077. The percent of P24Gag+ cells was normalized to the untreated condition for each virus (= 100%). (*p < 0.0001: p-values were calculated, between RPV treatment and no treatment for each TF virus, with an unpaired two-tailed t-test). Cell death was assessed 4 h after NNRTI treatment. **e** NNRTI resistance-conferring RT mutations in bold red type identified in pCH058 and PCH077 via the Stanford University HIV Drug Resistance Database [49–51]

NNRTI-resistant viruses with mutations in RT frequently emerge during therapy [24]. We therefore compared the effect of the well described RT mutation K103N [25] on both reverse-transcription inhibition and NNRTI-induced killing. With WT HIV-1, EC50 to inhibit infection was 0.15 nM for RPV and 0.2 nM for EFV (Fig. 4a). The RT K103N mutation only slightly altered susceptibility to RPV (EC50 = 0.3 nM), while the EFV dose response curve shifted 100-fold (EC50 = 20 nM). For NNRTI-induced killing of WT virus, RPV and EFV EC50 were 0.2 μM and 0.7 μM, respectively (Fig. 4b). The K103N mutation shifted the RPV concentration required for killing 4-fold (EC50 = 0.8 μM) while EFV was shifted more than 10-fold (EC50 out of range). This confirms that acquired NNRTI resistance also affects NNRTI-induced killing. The increased resistance of the K103N mutation to RPV in the context of NNRTI killing compared to RT inhibition could reside in the different nature of the target protein (GagPol polyprotein vs. mature RT).

HIV-1 infected individuals can also frequently acquire resistance to PIs; therefore, we examined the influence of PI resistance on PI inhibition of NNRTI killing. We tested two PI-resistant HIV-1 Isolates against three different PIs: IDV, saquinavir (SQV) and tipranavir (TPV) [26]. Maximum inhibition of WT HIV-1 was obtained with 1 μM IDV and SQV and 2 μM TPV (Additional file 1: Fig. S6). Using these concentrations, isolate 11803 was resistant to prevention of NNRTI-dependent killing by both IDV and SQV, but only marginally resistant to TPV (Fig. 4c). Isolate 11809 was primarily resistant to SQV but marginally resistant to IDV and TPV.

Both cytotoxic and anti-apoptotic effects have been described, respectively, for NNRTIs [27–29] and PIs [30–32] independent of HIV-1 infection. Importantly, our RT and PR resistance experiments indicate that both HIV-1 RT and PR are directly involved in NNRTI-induced killing and that there were no detected off-target effects of NNRTIs or PIs.

To further extend these findings with primary HIV-1 isolates, we tested a panel of Transmitter Founder (TF) viruses. Out of 9 TF viruses, 7 displayed an NNRTI killing response. Three were equally or more susceptible than the lab-adapted clone NL4-3, while 4 displayed intermediate phenotypes. Importantly, sequence analysis of the two TF viruses that were unresponsive to RPV revealed that they contained mutations predicted to confer RT resistance (Fig. 4e).

### Co-treatment with NNRTIs and latency reversing agents purges the latent provirus reservoir in vitro

**Fig. 5:**
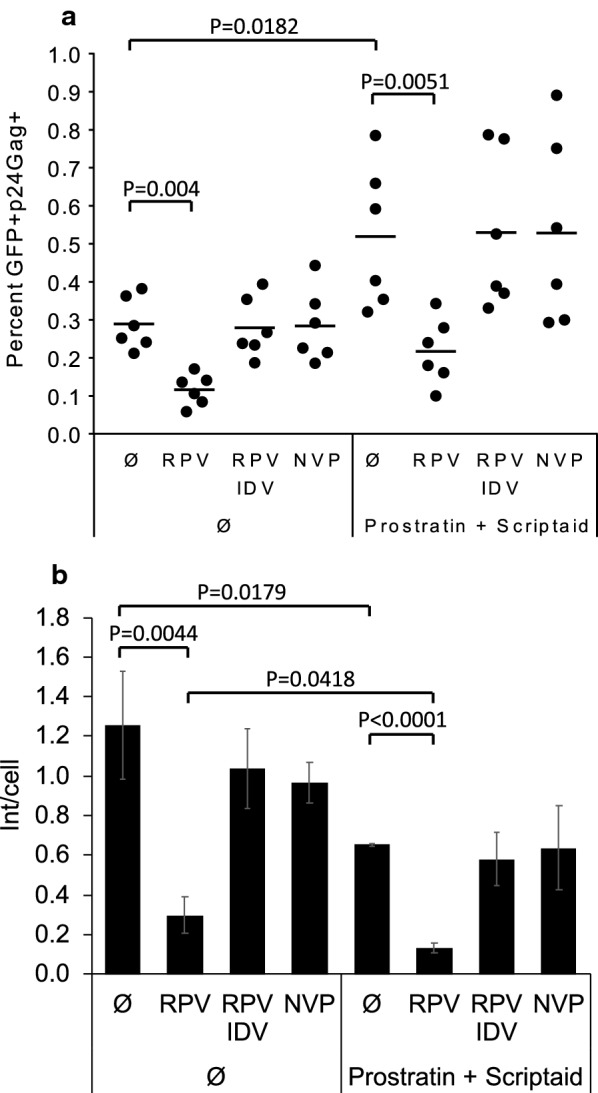
NNRTI-induced killing can be combined with LRA treatment to purge the latent reservoir. **a** IL-4 treated resting T cells were infected with a GFP reporter virus and cultured for 15 days. IL-4 was replenished on 7 dpi. Fifteen days after infection, GFP- cells were sorted and treated or not with combination of latency reversing agents (prostratin 330 nM and scriptaid 200 nM) plus RPV (1 μM), RPV + NVP (1 μM) or IDV (1 μM). Twenty-four hours later GFP and p24Gag expression were analyzed. Horizontal lines represent the mean of 6 donors from 2 independent experiments (*p = 0.0004; **p = 0.0182; ***p = 0.0051: p-values were calculated with an unpaired two-tailed t-test). **b** At 5 dpi, total infected cells were treated with a combination of LRA as in A. Twenty-four hours later dead cells were removed by Ficoll, DNA was extracted, and levels of provirus integration was measured by nested Alu-PCR. Data are averages from 3 donors. (*p = 0.0044; **p = 0.0179; ***p = 0.0418; ****p < 0.0001: p-values were calculated with an unpaired two-tailed t-test)

Finally, we tested the capacity of NNRTI-induced killing to kill latently infected CD4 T cells and purge integrated proviruses following virus reactivation. We infected resting peripheral blood CD4 T cells with an Envelope defective GFP reporter virus pseudotyped with HIV-1 Env [33]. After 14 days, GFP-negative cells containing latent virus were sorted, as previously described [18, 19]. Latent viruses were activated with a combination of prostratin and scriptaid [19], either with or without RPV. Even without stimulation, some spontaneous virus reactivation was observed, and RPV reduced the number of GFP+ p24Gag+ cells that emerged. Prostratin plus scriptaid increased virus expression two-fold over this background, and again, RPV reduced the number of emerging GFP+ p24Gag+ cells (Fig. 5a). Importantly, PI treatment prevented any cell loss.

In order to more accurately measure the effect of NNRTI and LRA combination treatment on the active and latent proviral reservoir, we measured the level of integrated HIV-1 genomes by Alu PCR. The same cotreatment as in Fig. 5a was performed on total infected cells at 5 dpi. Twenty-four hours later we removed dead cells by Ficoll gradient and measured the level of integrated provirus in the remaining live cells. As expected, RPV alone reduced the number of proviruses (Fig. 5b) in a PR-dependent manner. LRA treatment alone, which is toxic to productively infected cells, also contributed to reduce the number of proviruses. Interestingly PI treatment did not prevent this loss but rather only the NNRTI-induced cell proviral loss. Importantly, integrated proviruses were reduced further when RPV was combined with prostratin and scriptaid, indicating that latent proviruses were eliminated similarly to the sorted cells in Fig. 5a.

## Discussion

The Mueller laboratory first proposed the use of NNRTI-induced PR activation to eliminate infected T cells [15] following their observation that several days of NNRTI treatment resulted in a reduction in the number of productively infected cells. Here, we show that NNRTI-induced killing is highly efficient and rapid, leading to the elimination of HIV-1 expressing cells after only a few hours. These previously unreported rapid kinetics suggest in vivo feasibility of NNRTI as an adjuvant in the kill phase of shock and kill therapy. In NNRTI-treated patients, NNRTI plasma concentrations are in the range of efficient NNRTI-induced PR cytotoxicity reported here. For example, EFV remains above 3.2 μM (1 μg/mL) [34, 35] and RPV remains above 0.4 μM (400 ng/mL) [36]. However, lower penetration of NNRTIs occur in peripheral compartments such as lymphoid tissues, which are primary sites of the HIV-1 reservoir [37, 38]. These lower concentrations may not be adequate to support optimal NNRTI-induced killing. Therefore, treatment aimed at NNRTI killing may require a brief high dose in order to penetrate peripheral compartments. The rapidity of NNRTI-induced apoptosis we observe provides optimism that a single NNRTI dose might be effective. While excessive daily intake of NNRTI can be toxic [39, 40], a single high dose might be tolerated. The development of new agents specifically optimized for premature PR activation could aid the feasibility of this approach. Any ongoing PI therapy would clearly need to be stopped and PIs allowed to clear the system prior to such a treatment, owing to the inhibition of NNRTI-induced killing by this class of antivirals.

Using pan-caspase inhibitor Z-VAD-FMK we could only decrease and delay NNRTI-induced killing, suggesting that the activation of multiple pathways, both caspase dependent and independent, are involved in apoptosis induction here. Interestingly, the recently described pan-caspase inhibitor Q-VD-OPh was not able to inhibit NNRTI-induced cell death in our system (not shown). This fits well with previous reports which identified multiple host targets of PR activity relating to cell death [11–14]. We were surprised to see an inhibitory effect of Caspase 1 inhibitors. While HIV-1 infection has been linked to caspase 1 activation [41, 42], to our knowledge, no direct role of PR has been demonstrated. Alternatively, this may be explained by possible off-target effects [43, 44]. A more systematic analysis of target cleavage in this model will be necessary to better understand the precise cell death mechanisms involved.

In their study, Jochmans et al. noted that some NNRTI such as ETV and NVP did not induce PR activation [15]. In our study, all the NNRTIs tested had some ability to kill infected cells, albeit with very different efficiencies. This discrepency may result from a more sensitive detection of cell death in our system.

While the existence of ongoing replication of HIV-1 in ART treated patients is highly debated, much evidence supports the existence of persistent HIV-1 production in lymphoid tissue years after ART initiation at a time when virus RNA is below detection in the blood [45–47]. Such persistent virus producing cells could represent ideal targets for NNRTI-induced killing as they spontaneously produce GagPol proteins [47]. (It is unfortunate that PI drugs sometimes present in combination antiretroviral therapy would block this useful effect of NNRTI.) On the other hand, the reservoir of latently infected cells would be insensitive to NNRTI treatment and therefore require reactivation. Here, we have demonstrated that NNRTI-induced killing enhances the elimination of latently infected T cells following latency reversal. Interestingly, a recent study exploring the effect of antiretrovirals during virus reactivation also observed a reduction of both virus output and infected cells in the presence of NNRTIs EFV and RPV [16] at similar concentrations to those employed in the current study. The authors hypothesized that NNRTI-induced PR activation may explain their observations. Here we demonstrate that NNRTI-induced killing is immediately effective on cells bearing recently reactivated virus.

## Conclusions

HIV-1 PR cytotoxicity has been known since early in the epidemic. More than a decade ago, Tachedjian et al. first reported the capacity of NNRTIs to induce premature GagPol dimerization and PR activation. Studies from the Muller laboratory [15] and the Sluis-Cremer laboratory [16] proposed to harness this effect as a therapeutic approach to eliminate HIV-1 infected cells. The present work extends these finding to demonstrate that NNRTI-induced cell death is very rapid and robust, with the rapidity especially opening the possibility that short, high dose exposure may be a viable strategy to decrease the viral reservoir. The development of new drugs specifically designed to induce GagPol dimerization with low side effects seems a worthwhile pursuit. Screening for effectiveness of such compounds may be assisted by the highly sensitive methodology that we present here.

## Methods

### Viruses

Green fluorescent protein (GFP) reporter virus NLENG1-ES-IRES, and heat-stable antigen (HSA) reporter virus NLENHSA-ES-IRES have been previously described [18, 20]. Schematics of these reporter viruses are shown in Additional file 1: Fig. S1. Both were restricted to a single round of infection by two stop codons introduced into env after the vpu open reading frame [48]. Viruses were pseudotyped with HIV-1 NL4-3 Envelope by cotransfection of the reporter virus backbone plasmid with the NL4-3 env expression plasmid pCI-NLenv, as previously described [18]. pCI comprises the mammalian CMV promoter expression vector pCI (Promega) with the NL4-3 env open reading frame inserted into its multicloning site. RT mutant NLENG1-ES-IRES K103N was produced by substitution of the codon for lysine 103 in the RT gene by a codon for asparagine (AAA to AAC). HIV-1 infectious molecular clone pNL4-3 was obtained from the NIH AIDS Reagent Program (ARP) (Cat# 114). Virus stocks were generated by transfection of 293T cells using polyethyleneimine (PEI).

PR resistant HIV-1 clones (F190755_3, GQ213968, and V16970_2 GenBank KC109801; ARP Cat# 11803 and 11809, respectively) were obtained from the NIH ARP [26]. TF viruses were obtained from the and are part of panel of full-Length transmitted/founder HIV-1 Infectious molecular clones (NIH ARP Cat# 11919). Both PR resistant and TF viruses were pseudotyped with NL4-3 Envelope to facilitate infection of resting peripheral blood CD4 T cells.

### Cells

CD4+ T cells were isolated from HIV-negative donor peripheral blood buffy coats (New York Blood Center) by negative selection using the Dynabeads Untouched magnetic separation kit (Invitrogen) as previously described [18]. CD4+ T cells were typically ≥ 99% CD25− CD69− CD38dim HLA-DR− quiescent cells. CD4+ T cells were cultured at 2 × 10^6 cells per ml in Advanced RPMI 1640 (Life Technologies) with 10% fetal bovine serum (Atlanta Biologicals) plus 1% penicillin and streptomycin (Life Technologies), 1% l-glutamine (HyClone), and 50 μM β-mercaptoethanol (Sigma). IL-7 (2 ng/ml; BioLegend) or IL-4 (12.5 ng/ml; R&D Systems) was added once on the day of infection or as indicated. Where indicated, cells were activated with Dynabeads human T-activator CD3/CD28 (Invitrogen), per the manufacturer’s instructions, for the amount of time indicated. IL-2 (50 U/ml; obtained from NIH) was added 24 h after bead stimulation. Where indicated, dead cells were removed through Ficoll gradient (GE Healthcare). Where indicated, T cells apoptosis was induced by staurosporine (1 μM; BioVision). For latency reversal experiment, CD4 T cells were treated with Prostratin (330 nM, Santa Cruz Biotechnology) and Scriptaid (200 nM, Santa Cruz Biotechnology).

### Infections

Virus stocks were filtered (0.45 μm) and then treated with Benzonase (Novagen; 50 U/ml, 30 min, 37 °C) to eliminate residual plasmid DNA. Virus titers were determined by TaqMan reverse transcription-quantitative PCR (RT-qPCR) for HIV-1 RNA (target in integrase) [18] and normalized to 125–320 ng p24Gag equivalents per million cells. Infections were performed by spinoculation in the presence of 5 μg/ml DEAE-dextran (Sigma) for 2 h at 1200 × g and 37 °C [18]. T cells were then washed twice and placed back in culture.

### Non-nucleoside reverse transcriptase inhibitors (NNRTIs)

Efavirenz (EFV), rilpivirine (RPV), etravirine (ETV), nevirapine (NVP), dapivirine (DPV), doravirine (DOR), delavirdine mesylate (DLV) were used at 1 μM or as indicated.

### Protease inhibitors (PIs)

Indinavir (IDV, 1 μM), saquinavir (SQV, 1 μM), and tipranavir (TPV, 2 μM) were used at the minimum concentration achieving maximum NNRTI-induced killing inhibition in our system. All antiretroviral drugs were obtained from the NIH ARP.

### Staining for flow cytometry analysis

HSA surface expression was detected using APC anti-mCD24 (M1/69; BD Pharmingen). Annexin V (Invitrogen) staining was performed per manufacturer’s instructions with or without the presence of 7AAD (10 ug/mL; BD Pharmingen). Intracellular p24Gag staining was performed with phycoerythrin (PE)-conjugated HIV-1 p24Gag antibody (KC57-RD1; Beckman Coulter) with the a Cytofix/Cytoperm kit (BD Bioscience). DIOC6 (Enzo) staining was performed per manufacturer’s instructions. Briefly, cells were resuspended with 2 μM DIOC6 in serum free RPMI and incubated for 20 min at 37 °C, then washed three times with pre-warmed serum supplemented RPMI.

Flow cytometry was performed on a Becton–Dickinson FACSort flow cytometer upgraded with 3 lasers by Cytek Development, Fremont, CA, as previously described [18]. Compensation was applied during data collection on the basis of single-color controls. Flow data were analyzed with FlowJo software (version 9; Tree Star). Cell sorting was performed using a BD FACSAria at the New York University Langone Cytometry and Cell Sorting Facility.

### Alu PCR to measure integrated proviruses

DNA extraction and PCR to detect integrated genomes was performed as previously described [18].

## Additional file

**Additional file 1.** **Fig. S1**. Reporter viruses employed in this study. Diagram of the main characteristics of the reporter HIV-1 viruses employed in this study (see methods). **Figure S2.** Stability of GFP and HSA reporter proteins following apoptosis. T cells infected or not with GFP (NLENG1-ES-IRES) or HSA (NLENHSA-ES-IRES) reporter viruses were treated or not with staurosporine (1 μM) at 5 dpi. Twenty-four hours later GFP fluorescence and HSA expression (following anti-HSA staining) were measured by flow cytometry. **Figure S3.** Toxicity of NNRTI treatment on uninfected cells. Additional data from experiment in Fig. 1c, d here showing effects of NNRTIs on mock infected T cells. **Figure S4.** EFV killing of Infected resting T cells. Additional data from experiment in Fig. 2a showing killing with EFV. **Figure S5.** Addition of fresh IL-7 does not prevent NNRTI killing. IL-7 treated resting CD4 T cells infected with HSA reporter were treated at 5 dpi with EFV in the presence of freshly supplemented IL-7 or not. Cell death was analyzed by flow cytometry measuring FSC reduction of HSA+ cells after 24 h. Data are representative of 2 independent experiments. **Figure S6.** Inhibition of NNRTI killing by PIs IDV, SQV and TPV. T cells infected with NL4-3 virus were treated at 5 dpi with RPV for 4 h in the presence of various concentrations of the PIs IDV, SQV and TPV. Data represent the percentage of inhibition of RPV killing. Productively infected cells were identified by intracellular p24Gag staining. Data are representative of 2 experiments.

## Data Availability

All data generated or analyzed during this study are included in this published article and its supplementary information files.

## Abbreviations

NNRTI: non-nucleoside reverse transcription inhibitor
PI: protease inhibitor
RT: reverse transcriptase
PR: protease
HSA: heat stable antigen
EFV: efavirenz
RPV: rilpivirine
ETV: etravirine
NVP: nevirapine
DPV: darivirine
DOR: doravirine
DLV: delavirdine mesylate
IDV: indinavir
SQV: saquinavir
TPV: tipranavir
DPI: day post infection
GFP: green fluorescent protein
