# Supplementary material for: NNRTI-induced HIV-1 protease-mediated cytotoxicity induces rapid death of CD4 T cells during productive infection and latency reversal
Source: Retrovirology. 2019 Jun 26;16:17. doi: 10.1186/s12977-019-0479-9 (PMC6595680; doi:10.1186/s12977-019-0479-9)
Supplement: Supplementary file 1 — Additional file 1. Fig. S1. Reporter viruses employed in this study. Diagram of the main characteristics of the reporter HIV-1 viruses employed in this study (see methods). Figure S2. Stability of GFP and HSA reporter proteins following apoptosis. T cells infected or not with GFP (NLENG1-ES-IRES) or HSA (NLENHSA-ES-IRES) reporter viruses were treated or not with staurosporine (1 μM) at 5 dpi. Twenty-four hours later GFP fluorescence and HSA expression (following anti-HSA staining) were measured by flow cytometry. Figure S3. Toxicity of NNRTI treatment on uninfected cells. Additional data from experiment in Fig. 1c, d here showing effects of NNRTIs on mock infected T cells. Figure S4. EFV killing of Infected resting T cells. Additional data from experiment in Fig. 2a showing killing with EFV. Figure S5. Addition of fresh IL-7 does not prevent NNRTI killing. IL-7 treated resting CD4 T cells infected with HSA reporter were treated at 5 dpi with EFV in the presence of freshly supplemented IL-7 or not. Cell death was analyzed by flow cytometry measuring FSC reduction of HSA+ cells after 24 h. Data are representative of 2 independent experiments. Figure S6. Inhibition of NNRTI killing by PIs IDV, SQV and TPV. T cells infected with NL4-3 virus were treated at 5 dpi with RPV for 4 h in the presence of various concentrations of the PIs IDV, SQV and TPV. Data represent the percentage of inhibition of RPV killing. Productively infected cells were identified by intracellular p24Gag staining. Data are representative of 2 experiments. [file 12977_2019_479_MOESM1_ESM.pdf]

Figure S1

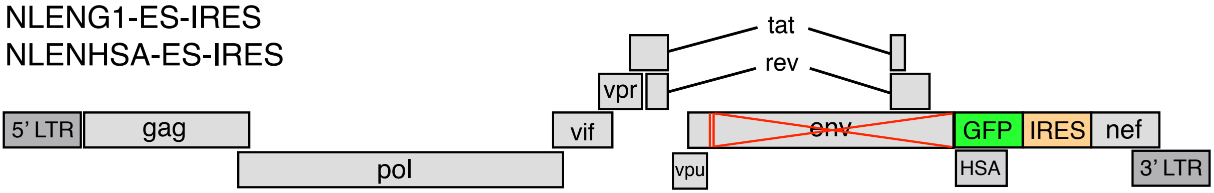
NLENG1-ES-IRES
NLENHSA-ES-IRES
tat
rev
vpr
5' LTR
gag
vif
env
GFP
IRES
nef
pol
vpu
HSA
3' LTR

Figure S2.

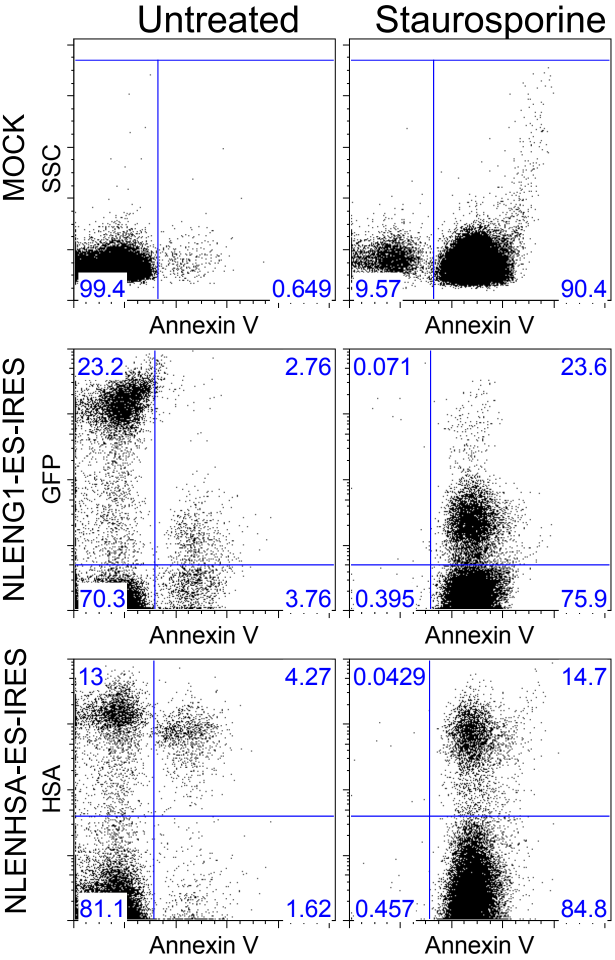

Untreated
Staurosporine
MOCK
SSC
99.4
0.649
9.57
90.4
Annexin V
Annexin V
NLENG1-ES-IRES
GFP
23.2
2.76
70.3
3.76
0.071
23.6
0.395
75.9
Annexin V
Annexin V
NLENHSA-ES-IRES
HSA
13
4.27
81.1
1.62
0.0429
14.7
0.457
84.8
Annexin V
Annexin V

Figure S3.

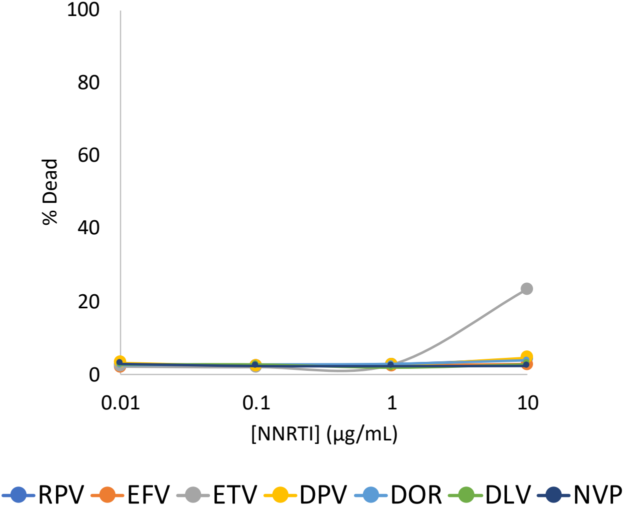

100
80
60
40
20
0
% Dead
0.01
0.1
1
10
[NNRTI] (μg/mL)
RPV
EFV
ETV
DPV
DOR
DLV
NVP

Figure S4.

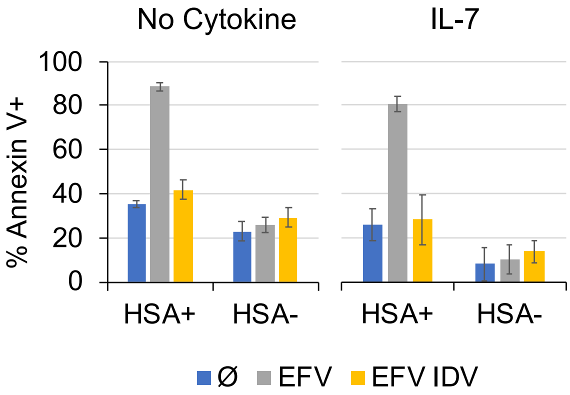
No Cytokine
IL-7
% Annexin V+
100
80
60
40
20
0
HSA+
HSA-
HSA+
HSA-
Ø
EFV
EFV IDV

Figure S5

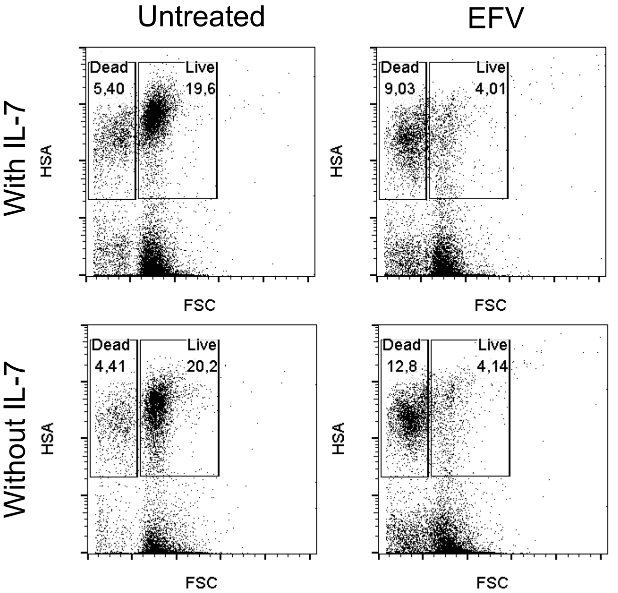
Untreated
EFV
With IL-7
Without IL-7
Dead
Live
5,40
19,6
9,03
4,01
4,41
20,2
12,8
4,14
HSA
FSC

Figure S6

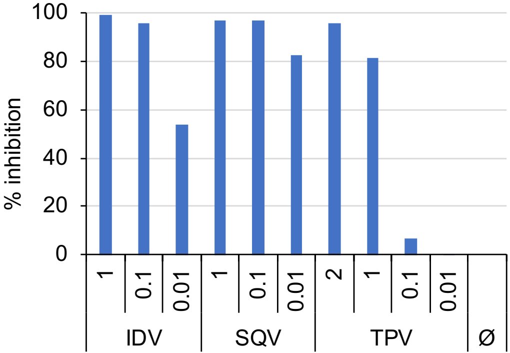

100
80
60
40
20
0
% inhibition
1
0.1
0.01
1
0.1
0.01
2
1
0.1
0.01
IDV
SQV
TPV
Ø
